# Site-Directed Mutants of Parasporin PS2Aa1 with Enhanced Cytotoxic Activity in Colorectal Cancer Cell Lines

**DOI:** 10.3390/molecules27217262

**Published:** 2022-10-26

**Authors:** Miguel O. Suárez-Barrera, Lydia Visser, Efraín H. Pinzón-Reyes, Paola Rondón Villarreal, Juan S. Alarcón-Aldana, Nohora Juliana Rueda-Forero

**Affiliations:** 1 Department of Pathology and Medical Biology, University Medical Center Groningen, University of Groningen, 9700 AB Groningen, The Netherlands; 2 Facultad de Ciencias Médicas y de la Salud, Instituto de Investigación MASIRA, Universidad de Santander, Bucaramanga 680002, Colombia; 3 Max Planck Tandem Group in Nanobioengineering, Institute of Chemistry, Faculty of Natural and Exacts Sciences, University of Antioquia, Medellín 050015, Colombia

**Keywords:** anticancer, parasporin, site-directed mutagenesis, apoptosis, APN receptor

## Abstract

Parasporin 2 has cytotoxic effects against numerous colon cancer cell lines, making it a viable alternative to traditional treatments. However, its mechanism of action and receptors remain unknown. In this study, site-directed mutagenesis was used to obtain PS2Aa1 mutants with variation in domain I at positions 256 and 257. Variants 015, 002, 3-3, 3-35, and 3-45 presented G256A, G256E, G257A, G257V, and G257E substitutions, respectively. Cytotoxicity tests were performed for the cell viability of cell lines SW480, SW620, and CaCo-2. Mutants 3-3, 3-35, and 3-45 efficiently killed the cell lines. It was found that the activated forms of caspase-3 and PARP were in higher abundance as well as increased production of γH2AX when 3-35 was used to treat CaCo-2 and SW480. To assess possible membrane-binding receptors involved in the interaction, an APN receptor blocking assay showed reduced activity of some parasporins. Hence, we performed molecular docking and molecular dynamics simulations to analyze the stability of possible interactions and identify the residues that could be involved in the protein–protein interaction of PS2Aa1 and APN. We found that residues 256 and 257 facilitate the interaction. Parasporin 3-35 is promising because it has higher cytotoxicity than PS2Aa1.

## 1. Introduction

The use of bacteria and their byproducts, i.e., attenuated or genetically modified, has begun to increase in recent decades based on their ability to recognize specific characteristics of cancer cells. They can act directly on mechanisms involved in the proliferation and growth of tumor cells [1]. Additionally, their use as antitumor agents represents a promising strategy because of the ease of their genetic manipulation, which allows biomolecules with improved toxic activity and specificity to be obtained [1,2,3,4]. Mizuki et al., in the first report on Parasporin (PS) in 2000 [5], described its potential as an anticancer molecule, specifically in leukemia. Subsequently, most studies have focused on the characterization and screening of new proteins identified from *Bacillus thuringiensis* (*Bt*) strains and isolates [6,7,8,9,10]. Akiba and Okumura proposed in 2017 that the mechanism of action of this protein is in inducing apoptosis [11], but many unknowns persist up to now, especially regarding the identity of the major PS receptor, with N-aminopeptidase (APN) and GPI-anchored proteins having been proposed as candidates [6,7,12,13]. Parasporin 2Aa1 (PS2Aa1) has shown cytotoxic effects against several human cancer cells, being highly specific for human liver and colon cancer cells [7,8,9], thus representing an additional treatment alternative for colorectal cancer. This protein is a polypeptide that requires proteases such as proteinase K to switch from a pro-toxin of 37 kDa to its active form of 30 kDa, which is shown to be highly cytotoxic and selective toward different cancer cell lines [10]. Furthermore, this protein is believed to act specifically on the membrane of target cells through domain I, which indicates the presence of a specific PS2Aa1 receptor whose identity is so far unknown [12]. Once PS2Aa1 is bound to the receptor, through domains II and III, it forms oligomers that permeabilize the membrane and lead to the formation of pores, which induces structural cytoskeletal alterations, organelle fragmentation, alterations in cell morphology, and finally lysis of susceptible cells [8]. Considering this scenario, our research group is studying the structure–function relationship of Parasporin 2, while exploring its possible use as a therapeutic alternative. This is how we recently designed and prepared peptides in which loop 1 of domain I was mutated, and the selection of this site in the design was supported by in silico modeling with APN. In that study, the Loop1–PS2Aa and P264–G274, peptides exhibited stronger anticancer activity than the wild type against SW480 and SW620, respectively, and demonstrated high effectiveness and selectivity; hence, they were proposed as possible alternative therapeutic agents for the treatment of colon cancer. In this sense, we decided to take domain I of PS2Aa1, mutate the residues that presented differentiated activities in the reported peptides, and consider additional positions for the modification of these peptides to generate the libraries of PS2Aa1 mutants, which we describe below [6]. The goal of this work was to obtain a characterized set of PS2Aa1 mutants with modifications in domain I and assess its cytotoxicity in colon cancer cells. Moreover, we used molecular docking and molecular dynamics simulations to analyze the possible interactions between PS2Aa1 and APN receptor, which have been proposed as a possible interacting protein. Additionally, our work contributes to the understanding of the relation between the structure and function of these proteins and their possibilities of being used as new treatments in the future.

## 2. Results

### 2.1. Cloning and Obtention of PS2Aa1 Mutants

**Figure 1:**
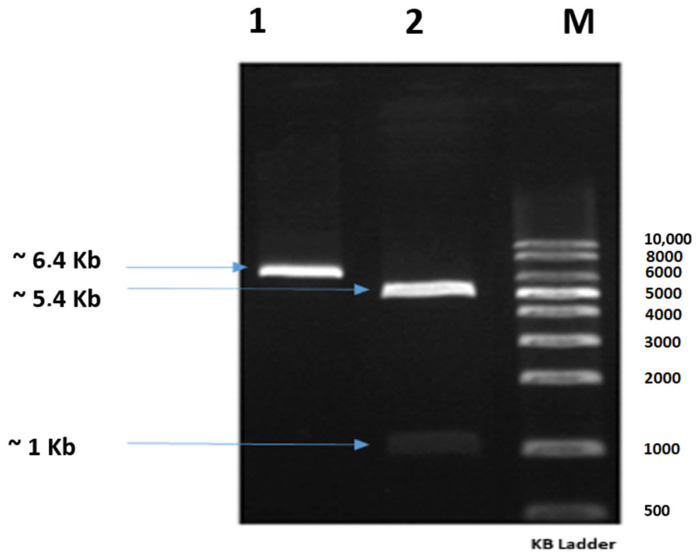
Agarose gel showing the construct *pET-30a/ps2Aa1* treated with the restriction enzymes *Kpn*I and *Hind*III; Lane 1: construct pET-30a/ps2Aa1; Lane 2: construct *pET-30a/ps2Aa1* treated with the restriction enzymes *Kpn*I and *Hind*III; Lane M: molecular weight marker.

The gene *ps2Aa1* (NCBI accession number AB099515.1) was cloned in the plasmid pET30a to generate the construct *pET-30a/ps2Aa1*, which was corroborated by restriction assays with a band of ~1 kb for PS2Aa1 and a band of ~5.4 kb for pET30 (Figure 1). Then, the construct was transformed into *E. coli* strain DE3BL21 for the production of plasmid and subsequent site-direct mutagenesis to create four libraries, one for each primer set.

A low mutation rate was obtained, whereby only 12 of the 71 selected mutants had mutations in their sequences. Therefore, from the 12 PS2Aa1 mutants, 6 were chosen for cytotoxicity assays. Mutant 0-2 presented an amino acid change from glycine (G) to aspartic acid (D) at position 256 of its amino acid chain corresponding to position 1000 of the nucleotide sequence; mutant 0-6 had an amino acid change from glycine to valine (V) in the same position; mutant 0-15, like the previous proteins, presented an amino acid change at position 256 to become alanine (A); therefore, these three mutants were obtained from the primer set flanking nucleotide position 1000. On the other hand, mutants 3-35 and 3-45 presented amino acid changes in their sequences at position 257 (position 1003 in the nucleotide chain), changing from glycine (G) to valine (V) and glutamic acid (E), respectively. It is, therefore, assumed that these mutants were obtained from the primer set targeting position 1003.

### 2.2. Description of Parasporins Obtained from PS2Aa1 Using Site-Directed Mutagenesis

From the libraries in *E. coli*, 103 colonies were sequenced. Mutagenesis was successful, although not in all the colonies because of the randomness attributed to the reaction by adding equimolar conditions of adenine, guanine, cytosine, or thymine.

The sequence analysis showed 99.9% identity with parasporin 2 of *Bt*, determined by BLASTn searching of the contigs for each variant, and MatGat, similarity/identity matrices for DNA [14]. The contigs were translated to their respective amino acid sequence and a multiple sequence alignment was performed comparing each of these sequences with the native parasporin reported in the PDB (code: 2ztb). The substitutions obtained at residues 256 and 257 were mostly glycine for alanine (A), aspartic acid (D), valine (V), and glutamic acid (E), as described in Table 1.

### 2.3. Cytocidal Activities of PS2Aa1 Variants in Human Colorectal Cancer Cells

We first compared the native *Bt* protein 4R2 with the recombinant PS2Aa1 and found that the activities were comparable in SW480 and SW620, but PS2Aa1 was more effective than 4R2 in CaCo-2 (Figure S1). Cell line NCM460 was used as a control. In this normal colorectal cell line, none of the parasporins showed any relevant effect, consistent with previous reports of specificity of the parasporin to cancer cells (Figure S1). The variants showed different patterns in the cell lines. In SW480, the protein with the highest cytotoxic activity was 3-35 with an IC_50_ of 0.32 µg/mL, more than three times that of PS2Aa1 (IC_50_ = 1 µg/mL). All of the variants were effective, with 002 having the lowest cytotoxicity (IC_50_ = 4.68 µg/mL). In SW620, 3-35 was also the most cytotoxic (IC_50_ 2.06 µg/mL), while 3.45 was the least (IC_50_ 17.77 µg/mL). Against CaCo-2 cells, the mutant 3-35 was the most cytotoxic (IC_50_ 0.96 µg/mL), with similar results observed for 3-3 and 3.45, whereas 0015 and 002 were not effective (Table 2).

**Figure 2:**
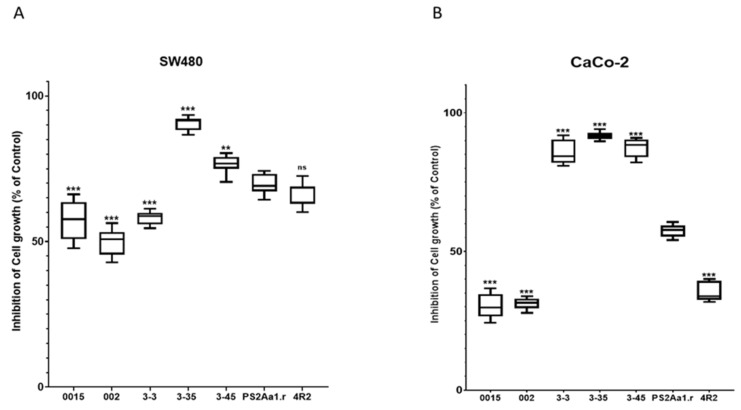
Tukey’s multiple comparison test for (**A**) SW480 and (**B**) CaCo-2. The results for the percentage of cell growth inhibition correspond to 5 µg·mL^−1^ for each parasporin. PS2Aa1 was used as a control. ** *p* ≤ 0.01, and *** *p* ≤ 0.001, ns (non-significant).

Comparisons with PS2Aa1 were based on ordinary one-way ANOVA employing Tukey’s multiple comparison test for SW480 and CaCo-2. All the comparisons were significantly different (**, ***), except between the activity of PS2Aa1 and the native strain 4R2 in SW480 (Figure 2).

### 2.4. Variants of PS2Aa1 Induce Apoptosis in Human Colorectal Cancer Cells

To expand our results, we performed an analysis with annexin V (AV) and propidium iodide (PI) to characterize the percentage of cells in apoptosis and death. For this part of the study, the cell lines SW480 and CaCo-2 and the parasporins 015, 3-35, and PS2Aa1 were selected, owing to the observation of relevant cytocidal activity.

**Figure 3:**
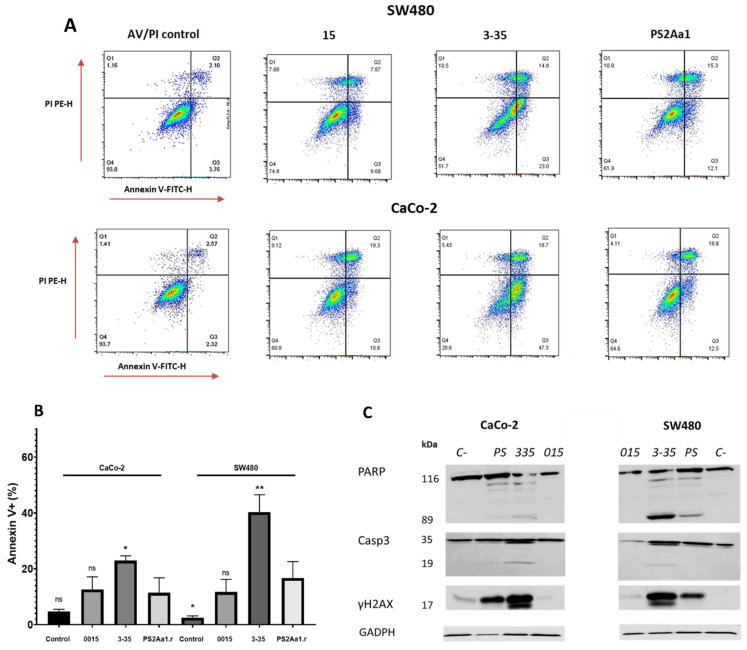
Cytotoxic effects of parasporins to CaCo-2 and SW480. Detection of annexin V/PI (**A**), statistical analysis of induction of apoptosis by parasporins (**B**). Western blot of PARP, caspase-3 (Casp3), and γH2AX in SW480 and CaCO-2 after treatment with the indicated parasporins (**C**). GAPDH was used as a loading control. The amount of toxins used for each treatment was 5 ug/mL over 48 h. * *p* ≤ 0.05, ** *p* ≤ 0.01, ns (non-significant).

After the treatment of CaCo-2 and SW480 with 5 µg/mL of each parasporin, higher percentages of apoptotic cells, 25% and 37%, respectively, were observed for both cell lines when treated with parasporin 3-35 followed by PS2Aa1 and, lastly, 015 (Figure 3A,B), which is similar to the cytotoxicity results, where 3-35 also excels. The differences between PS2Aa1 and 015 were not remarkable, and no differences in dead cells were detected in all of the treatments, suggesting the late activation of apoptosis in the cell lines by parasporin 2Aa1 and its variants as an effect of the pore-forming action of these proteins, which was previously suggested to be the mechanism of action [13,15,16,17]. HSD Tukey testing was performed with the results of annexin V-positive cells, showing that there are significant differences between PS2Aa1.r and 3-35 in the treatment of CaCo-2 (*). For SW480, it was shown that there are differences between PS2Aa1.r and 3-35 (**) and Ps2Aa1.r and the control (*); *p* ≤ 0.05 (Figure 3B).

To further investigate the induction of apoptosis, we also measured different markers using Western blot analysis, such as cleaved caspase-3, cleaved poly (ADP-ribose) polymerase-1 (PARP), and Histone 2 family member, phosphorylated on serine 139 (γ-H2AX). For both CaCo-2 and SW480, activation of caspase-3 and PARP was more induced by the treatment with parasporin 3-35, with multiple bands obtained for both proteins. PARP cleavage is more pronounced in SW480 and corresponds with the lowest IC_50_ (0.32 µg·mL^−1^) in the cytotoxicity assay (Table 2). We did not see differences between the results of caspase-3 and γH2AX in the cell lines.

In the case of γH2AX, the band intensity in the treatment with 3-35 is the highest. The presence of a slightly higher molecular weight band at 17 kDa indicates the phosphorylated form of this modified histone involved in the repair of DNA damage (Figure 3C).

### 2.5. Cytotoxic Activity of PS2Aa1 Variants Is Affected by APN Receptor Inhibition

**Figure 4:**
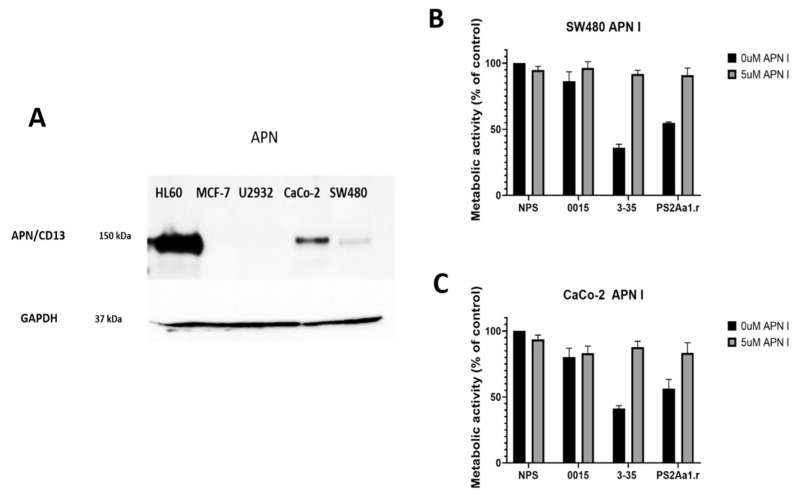
(**A**) Detection of APN receptor in different cell lines HL60, MCF-7, U2932, CaCo-2, and SW480. Metabolic activity of (**B**) SW480 and (**C**) CaCO-2 without parasporin (NP), parasporin 0015, 3-35, and the recombinant of PS2Aa1 (PS2Aa1.r) at 5 µg·mL^−1^ in the presence or absence of the APN inhibitor.

We next checked for the presence of APN as a possible receptor for PS2Aa1. The highest level of expression of the APN receptor was obtained in the positive control cell line HL60, and the protein was not present in negative controls MCF-7 and U2932 (Figure 4A). In the case of colorectal cancer cells, the amount of APN is higher in CaCo-2 compared with SW480. We next blocked APN with an inhibitor and tested the effect of the different parasporins at 5 µg·mL^−1^. The effect of 3-35 and PS2Aa1 on SW480 and Caco-2 was diminished to the point of no cytotoxicity at the lowest concentration of APN inhibitor (5 µM) (Figure 4B,C), which strongly suggest that the effect of 3-35 and PS2Aa1 is presumably dependent on binding to APN. To further understand these results, in silico analysis was performed via docking and molecular dynamics simulations.

### 2.6. Molecular Docking and Molecular Dynamics Analysis Highlight Residues 256 and 257 of Domain I of PS2Aa1

**Figure 5:**
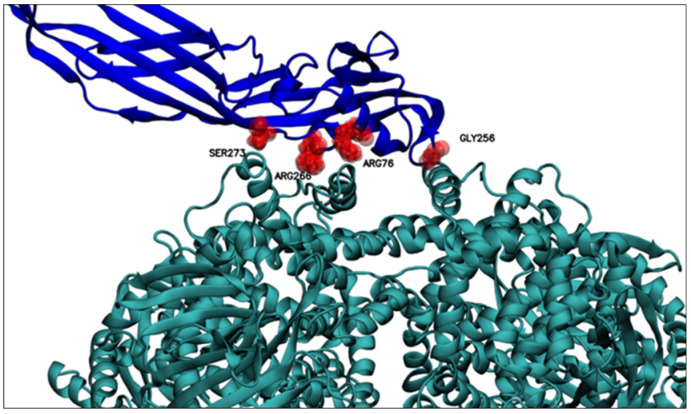
Relevant residues for PS2Aa1–APN interaction. Relevant residues for interacting with the APN receptor are displayed. GLY256, ARG76, ARG266, and SER273. PS2Aa1 and APN are colored blue and teal, respectively.

The predicted models were ranked based on the number of hydrogen bonds in the interface, with the top 10 shown in Table 3. After visual inspection, model number 560 was selected to perform the molecular dynamics simulations. Figure 5 shows the selected complex between PS2Aa1 and APN with the relevant residues in the interaction.

The 560-model selected by molecular docking analysis was subjected to molecular dynamics computational simulations to identify possible residues of wild-type PS2Aa1, which showed preference in protein–protein interaction (PS2Aa1–APN). As shown in Table 4, after three molecular dynamics simulation replicates, some of the PS2Aa1 residues were in contact with APN for the longest simulation time and at a smaller average distance from the center of mass of the PSAa1 residue and the closest APN residues. Table 4 records the wild-type PS2Aa1 residues, for each of the three simulation replicates, that were in contact for more than 80% of the simulation time and that also maintained inter-action distances of less than 5 Å. These two conditions suggest in silico that these residues could be of interest in the PS2Aa1–APN interaction.

**Figure 6:**
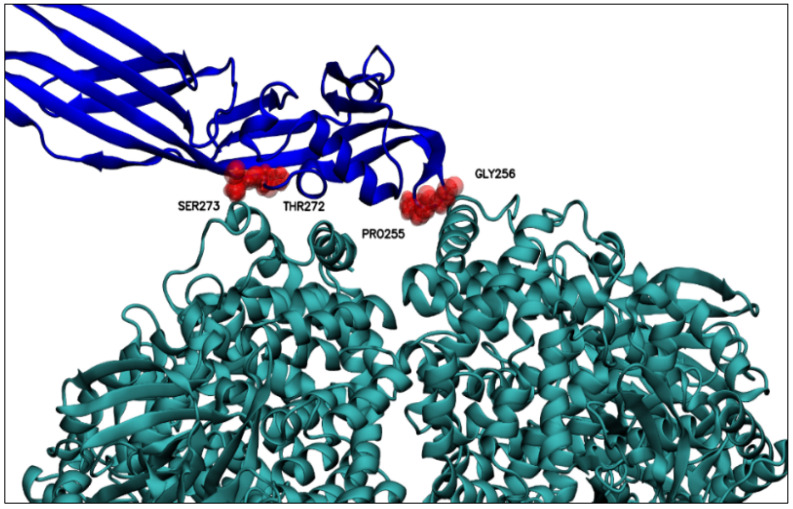
Residues relevant for the interaction with APN at distances less than 6 Å between the center of mass of the relevant PS2Aa1 residues and the nearest APN residue. Above, PS2Aa1 and APN are colored blue and teal, respectively.

Table 5 below presents a review of these residues from their prevalence in the three replicates, that is, the frequency with which these residues were identified as preferential for interaction in the molecular dynamic simulations (Table 4). There, GLY256 seems to be the residue of conspicuous importance, as it is the only residue recorded in all three replicas of molecular dynamics; the importance of this residue could be explained based on its position, because GLY256 is in a PS2Aa1 loop (Figure 5), a mobile and flexible part of the protein. Moreover, GLY256 presents stable dynamics (SD 0.21 Å), with an average minimum distance of 4.44 Å to APN (Figure 6), and is the closest residue.

Residues ARG76, PRO238, ILE239, THR240, VAL241, PRO255, GLY257, ARG266, THR272, SER273, and GLY274 had high prevalence in two MD replicates (Table 6), high-lighting the significant prevalence (frequency) of residues ARG76, ARG266, and SER273 that were close to the APN receptor. These three residues are found within the beta-sheet secondary structure of PS2Aa1 (Figure 5), which could limit their interacting with APN.

There are also some residues that could have had a modest role in the PS2Aa1–APN interaction because they appeared in only one MD replicate (ASP267, ASN270, and THR275) and were the farthest residues from APN. Likewise, the previous group of residues were located in the beta-sheet secondary structure of PS2Aa1, which reduced their chance to interact with APN.

Lastly, some high-prevalence residues have an average distance of less than 6 Å (Table 6). These were PRO255, GLY256, THR272, and SER273, with GLY256 having the shortest distance and significant relevance in all the simulations (Table 6, Figure 6).

These results also suggest the participation of amino acids PRO255 and GLY256 as part of the loop of domain I of PS2Aa1 in the interactions with the APN receptor (Figure 6), being within the top 4 of the residues with the shortest interaction distance (Table 6).

## 3. Discussion

In this study, genetic modification was used to obtain mutants with substitutions in residues 256 and 257 of PS2Aa1. These modifications allowed us to build a library of new parasporins with different activities against colorectal cancer cell lines. Site-directed mutagenesis was performed considering the results of Cruz et al. [6], where peptides from loop 1 of PS2Aa1 had remarkable activity and adherence with SW480. It was established that the oligonucleotides with the mutations incorporated for site-directed mutagenesis should be present at the N-terminal end (variable region), specifically in domain I of PS2Aa1 because, based on previous molecular docking studies, this region is the one presumably responsible for specific binding to membrane receptors [14]. The search for conserved domains found that PS2Aa1 shares conserved domains with aerolysin-type βPFT proteins, which comprises a highly conserved region corresponding to the chain of the C-terminal end and a highly variable region at the N-terminal end of the protein, which usually contains recognition signals [11] and corresponds to domain I of the βPFT proteins. Its high variability means that this family of proteins has various binding receptors that are highly specific.

For the mutants, we obtained a selectivity index of 18.9 and 17.5 for PS2Aa1 against SW480 and SW620, respectively, and 46.4 and 34.4 for variant 3-35 against SW480 and SW620, respectively (data not shown). PS2Aa1 is described as an aerolysin and beta-pore-forming protein type because of their shared homology [15]. Parasporins and aerolysins are anticancer proteins [16] and the cytogenetic effects of aerolysin produced by *Aeromonas hydrophila* on normal and tumor cells have been studied; however, the latter present a toxic effect upon normal cell lines, unlike parasporins, which have an undetectable or only slight effect on normal cell lines [7,8] and a greater effect on several cancer cell lines.

Concerning the IC_50_, 40.15 μg·mL^−1^ PS2Aa1 and 39.93 μg·mL^−1^ 3-35 were noted for CHOK-1 and >100 μg·mL^−1^ for both against NCM460 presenting the parasporins and their variants as potential candidates for cancer treatment in the future. Moreover, in this study, we decided to analyze the possible interaction between PS2Aa1 and the designed mutants with the membrane receptor APN. Our results were like those found by Periyasamy et al. (2016) [7], where the use of an APN inhibitor reduced the cytotoxic activity of PS2Aa1. We found that the lowest concentration of APN inhibitor led to the loss of the cytotoxic activity of 3-35 and PS2Aa1 with CaCo-2 and SW480. Although not conclusive, our results showed that variant 3-35 requires, to some extent, APN receptor for its cytotoxic activity against Caco-2 and SW480. However, it is interesting to note that the mutant 3-35 achieved the highest cytotoxicity with cell line SW480, despite the CaCo-2 cell line having higher amounts of APN. Hence, it is likely that APN is not the only receptor directly involved in the mechanism of action of these toxins [6,17]. As previously mentioned, GPI-anchored proteins play a crucial role in the interaction with the cell membrane. These receptors seem to be more relevant in the activity of the PS in cancer cell lines because cell lines such as MCF-7 lack APN, but PS2Aa1 has strong activity against this line [18]. It has been suggested that APN could be a receptor in the mechanism of action of PS2Aa1 because parasporin activity decreased substantially in HCT116 cells, a colorectal cancer cell line, when treated with an APN inhibitor [7]. In addition, it is known that PS2Aa1 is related to the Cry proteins, which use APN as a receptor in the midgut of insects [17,19,20,21,22,23]; in this scenario, APN receptor is important for parasporin activity. The data indicate that, although there was a decrease in cytotoxic activity, it was not completely lost, which leads to the assumption that APN is important, but it is not the only receptor involved. Considering another binding receptor for PS2Aa1, the GPI-anchored proteins have also been suggested as relevant to the binding to the cell membrane. It was determined that the glycan core, which is part of CD59, is essential for the recognition of the aerolysin-type parasporin [6,7,12].

PS2Aa1 in its native form can induce apoptosis in mammalian cancer cells [24] like PS produced by *B. thuringiensis* A1519, which induces the mitochondrial apoptosis pathway in Jurkat cells via caspase-3 and -9 cleavage followed by the release of cytochrome C [25]. In this study, we showed through cytotoxicity assays that the mutant 3-35 had a more remarkable effect on cancer cell lines, and the amount of cleaved caspase-3 and PARP and the production of γH2AX were reported for the first time. We also presented the effect of this mutant in early and late apoptosis with respect to recombinant PS2Aa1; these results suggest that the point variation G257V on Loop 1 can alter the behavior of the protein measured by its differentiated cytotoxicity and apoptosis induction when compared with the native PS2Aa1.

**Figure 7:**
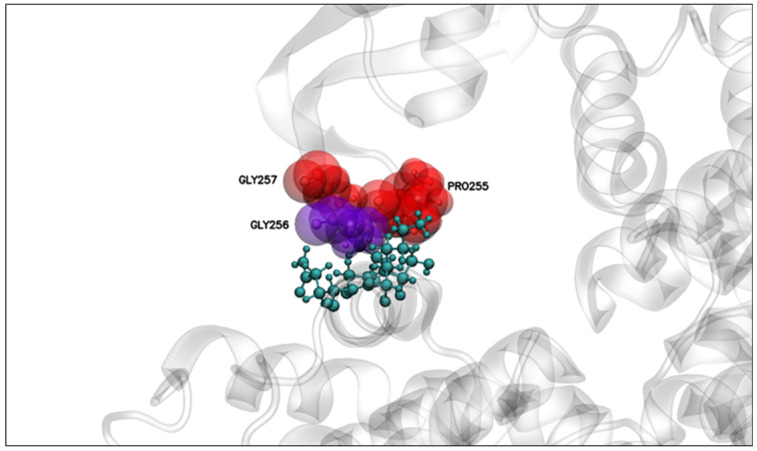
PS2Aa1–APN interaction. Relevant residues for interaction with APN are analyzed in Figure 6. Distances were computed between the center of mass of the relevant residues of PS2Aa1 and the closest residue from APN.

The molecular docking and dynamics simulations showed that it is likely that a stable interaction between domain I of PS2Aa1 and APN receptor could take place. Moreover, the mutation of mutant 3-35 was performed in the region of amino acids involved in interactions during the molecular dynamics simulations (Figure 7). Residue 256 was present in all three replicates, with the smallest interaction distance between its center of mass and the APN contact surface (Figure 7). The experimental results showed that its mutation reduced the interaction and cytotoxicity, so the G in position 256 seems relevant for the cytotoxic effects of PS2Aa1. In contrast, the mutation of position 257, which was involved in two replicates of molecular dynamic simulation, led to an improved interaction and cytotoxicity, i.e., the change of glycine for valine improved the cytocidal activity of the designed mutant 3-35 by creating a more nonpolar amino acid, suggesting that it feasibly conferred more stability on the protein. This mutant outperformed native Ps2Aa1 and 4R2 in terms of cytotoxicity in all three tested cell lines: SW480, SW620, and CaCo-2.

Finally, the use of site-directed mutagenesis might helpful to obtain a clearer understanding of how parasporin 2 works and to shed some light on understanding the action mechanism while generating proteins with enhanced activity like variant 3-35. This study reveals interesting features of domain I and highlights the relevance of residues 256 and 257 in the interaction with possible receptors that might be interacting with PS2Aa1, such as APN, and membrane-anchored receptors or GPI-anchored proteins [12].

## 4. Materials and Methods

### 4.1. Bacterial Strains and Culture Conditions

*E. coli* strain DE3BL21 harboring the construct pET30a + PS2Aa1 and *Bacillus thuringiensis* (*Bt*) BMB171 were cultured in Luria Bertani (LB) broth incubated at 37 °C with constant agitation for 24 h.

### 4.2. Parasporin Site-Directed Mutagenesis

Mutagenesis assays were carried out using the Gene-Art Site-Directed Mutagenesis Plus kit according to the manufacturer’s instructions and four sets of designed primers (Table 7). Reaction and amplification conditions were developed according to the kit specifications.

Each of the mutation reactions was directly transformed into chemically competent TOP10F and DH5αT1 cells, 70 µL of the transformed cells was then inoculated on plates with LB + kanamycin agar (25 µg/mL) + X-gal + Isopropyl β-D-1-thiogalactopyranoside (IPTG) and incubated for 18 h at 37 °C, and white/blue cell screening was performed.

### 4.3. Library Verification

The positive clones (blue colonies) were cultured in LB plates with kanamycin (25 µg/mL). A single colony was selected and cultured in 5 mL LB broth at 37 °C overnight with agitation at 200 rpm. The plasmid pET-30PS2-Variant was isolated using the Wizard Plus SV Minipreps DNA purification system (Promega^®^, Madison, WA, USA) according to the manufacturer’s instructions. Sanger sequencing of the desired fragments using universal T7 primers was performed by Macrogen^®^ (Seoul, Korea). The ~1000 bp contig was assembled using DNA Baser version 5.15 (Sequence assembly software) and used in BLASTn search (National Center for Biotechnology Information; www.ncbi.nim.nih.gov accessed on 19 September 2022). The protein sequence was deduced using the Translate tool available at Expasy (https://web.expasy.org/translate/, accessed on 20 September 2022) followed by multiple alignment sequence analysis of the variants in comparison with native PS2Aa1 using Bio-Edit Software [26]. Finally, variants with non-silent mutations were transformed into *Bt* BMB171 via electroporation using two consecutive electric pulses of 1.5 kV during 4.5 ms. Following this procedure, four PS2Aa1 mutant libraries were created.

### 4.4. Preparation of Activated Parasporin Proteins

*Bt* BMB171 variants were cultivated in LB broth and incubated for 5 days at 30 °C. The cells were then harvested by centrifugation at 10,000 rpm for 10 min; the pellet containing the precipitated parasporin proteins was solubilized in 5 mL of solubilization buffer (56 mM Na_2_CO_3_ (pH: 11.4) and 11 mM of dithiothreitol (DTT) (pH: 11.4)) for 2 h at 37 °C. Insoluble material was removed by centrifugation at 10,000 rpm for 10 min and the supernatant was passed through a 0.22 μm membrane filter. The filtrate (70 mL) pH was adjusted to 8 using 1 M Tris-HCl (pH 8).

The solubilized proteins were digested using proteinase K (final concentration of 185 μg/mL) for 1 h at 37 °C. Phenylmethylsulfonyl fluoride (PMSF) was added (final concentration 1 mM) to stop proteolytic processing. To confirm the presence of the parasporin proteins, SDS–PAGE analysis was performed as previously described [19]. The protein concentration was determined using the Bio-Rad Protein Assay (Bio-Rad Laboratories, Mississauga, ON, Canada).

### 4.5. Colon Cancer Cell Lines

Colon cancer cell lines were obtained from the American Type Culture Collection (ATCC). Lines SW-480 and CaCo-2 were cultured in Dulbecco’s modified Eagle’s medium (DMEM) with 25 mM glucose and 2 mM L-glutamine, supplemented with 10% fetal bovine serum (FBS), 10,000 µg/mL penicillin and streptomycin, and 1% non-essential amino acids; SW620 and NCM460 were cultured in RPMI supplemented with 10% FBS. The cultures were incubated in a humidified incubator at 37 °C under a 5% CO_2_ atmosphere.

### 4.6. Cytotoxicity Assays of PS2Aa1 Mutants in Colon Cancer Cells

To characterize the anti-proliferative activity of the toxins PS2Aa1 and mutants, concentrations ranging from 0.25 to 5 μg/mL of the activated protein were prepared. The cells were incubated with parasporin for 72 h, and anticancer activity was assessed on the basis of cell viability following incubation with alamar blue (BioRad, Hercules, CA, USA) for 5 h at 37 °C under a 5% CO_2_ atmosphere. Emission at 560 nm and excitation at 590 nm were measured using a CLARIOstar reader. IC_50_ concentrations were calculated from these data.

### 4.7. APN Detection and Blocking Assay

Western blot detection of APN was carried out using antibody CD13/APN (Cell Signaling Technology^®^, Beverly, MA, USA) and GAPDH was used as a loading control. The proteins extracted from HL60 and MCF-7 were used as the positive and negative control, respectively. The blocking assay was performed as reported by Periyasamy et al. 2016 [7]. Briefly, CaCo-2 and SW480 cells were used, but, because of the presence of APN receptors, the concentration of the APN blocker Dinitroflavone (Santa Cruz Biotechnology, Inc., Dallas, TX, USA) was diluted from 5 to 50 μM and added to the cells, with triplication of samples. This was followed by incubation for 72 h at 37 °C under a 5% CO_2_ atmosphere using 3 µg of parasporin. The results of metabolic activity were analyzed using the alamar blue assay, as previously described [7].

### 4.8. Molecular Docking and Molecular Dynamics Analysis

Molecular docking analysis was performed to determine possible interactions between PS2Aa1 and the APN receptor. The 3D structures of PS2Aa1 and APN were downloaded from the Protein Data Bank with PDB ID 2ZTB and 6ATK, respectively. The simulations were performed using the protein–protein global docking protocol of Rosetta [27] with flags construct-5000, -spin, -randomize1, and -randomize2, and simulations were run in the software version 3.9. Later, the Interface Analyzer protocol of Rosetta [28] was used to analyze the interaction between PS2Aa1 and APN. Next, the top 10 models were visually inspected for the selection of a suitable model in which the amino acids corresponding to domain I of PS2Aa1 were interacting with a valid region of APN. Subsequently, the selected model was used to perform molecular dynamics (MD) simulations, in triplicate, for PS2Aa1–APN complexes. The MD simulation protocol included solvation with TIP3P water molecules, and Na^+^ and Cl^−^ ions were added to ensure the neutrality of the system at an ion concentration of 150 mM. The Amber ff19SB force field for proteins was implemented for all systems.

The system was first minimized and equilibrated using Amber18 software. For the parasporin–APN complexes, stepped minimization, heating, and balancing were performed. Complexes were first minimized for 5000 (steepest descent) and 10,000 (conjugate gradient) followed by heating for 1 ns and then in an NVT to 300 K. For all simulations, an 8 Å cutoff was used for unbounded Coulombic and Lennard–Jones interactions and for periodic boundary conditions with a particle mesh Ewald treatment of long-range Coulombic interactions. A 2 fs time-step was employed by the SHAKE algorithm and the production steps were 100 ns for both systems.

### 4.9. Statistical Analysis

Type 1 ANOVA and Tukey’s test was performed using Graphad Prism 8. Statistical significance was indicated as * *p* ≤ 0.05, ** *p* ≤ 0.01, and *** *p* ≤ 0.001, with an IC of 95%.

## Figures and Tables

**Table 1:** Description of parasporins obtained from PS2Aa1 using site-directed mutagenesis.

ID (Library)	Type/Site of the Mutation	Mutant Name	Amino Acid Sequencing
Wt.	PS2Aa1 (PDB 2ZTB)	-	251-KRVGP**GG**HYF-260
02	SUS/256	G256D	251-KRVGPDGHYF-260
015	SUS/256	G256A	251-KRVGPAGHYF-260
3-3	SUS/257	G257A	251-KRVGPGAHYF-260
3-35	SUS/257	G257V	251-KRVGPGVHYF-260
3-45	SUS/257	G257E	251-KRVGPGEHYF-260

**Table 2:** Cytocidal activities of PS2Aa1 variants in human colorectal cancer cells.

Parasporin	IC50 Parasporin µg·mL^−1^ (95% CI)		
	SW480	SW620	CaCo-2
0015	2.42 (1.59–4.70)	ND	ND
002	4.68 (2.57–17.74)	ND	ND
3-3	2.02 (1.51–2.96)	ND	0.98 (0.83–1.16)
3-35	0.32 (0.22–0.43)	2.46 (2.07–3.00)	0.88 (0.81–0.95)
3-45	0.78 (0.65–0.92)	ND	0.96 (0.84–1.09)
P2SAa1	1.00 (0.65–1.55)	5.70 (4.10–9.50)	2.57 (1.84–4.15)
4R2	0.62 (0.41–0.86)	ND	ND

ND: Non-detectable.

**Table 3:** Top 10 models according to the number of hydrogen bonds in the interface.

Top	Model	Number of Hydrogen Bonds in the Interface
1	560	9
2	3413	9
3	105	9
4	819	8
5	994	8
6	2079	8
7	3521	8
8	1015	8
9	1742	8
10	708	8

**Table 4:** Residues in contact with APN receptors.

MD	Contact Residues (>80%) *
Replicate 1	PRO255, GLY256
Replicate 2	ARG76, PRO238, ILE239, THR240, VAL241, ARG266, GLY256, GLY257, THR272, SER273, GLY274
Replicate 3	ARG76, PRO238, ILE239, THR240, VAL241, ASP242, PRO255, GLY256, GLY257, ARG266, ASP267, ASN270, THR272, SER273, GLY274, THR275

* The residues in contact with APN receptors at 80% frequency are shown for each independent MD.

**Table 5:** Prevalence of PS2Aa1 residues among MD replicates.

MD	PS2Aa1 Residues
Replicate 3	GLY256
Replicate 2	ARG76 *, PRO238, ILE239, THR240, VAL241, PRO255, GLY257, ARG266 *, THR272, SER273 *, GLY274
Replicate 1	ASP267, ASN270, THR275

* Residues with the highest frequency in the MD replicates.

**Table 6:** Distances of the residues of PS2Aa1 relevant to interacting with APN.

Comparison	Mean	SD
GLY256	4.44	0.21
THR272	5.53	0.61
SER273	5.58	0.09
PRO255	5.77	0.26
THR240	6.04	0.27
ARG266	6.29	0.70
PRO238	6.30	0.58
GLY274	6.33	0.23
ASP267	6.49	0.44
ARG76	6.58	0.55
GLY257	6.64	0.69
ASN270	7.04	0.79
THR275	7.59	0.45
VAL241	7.64	0.75
ILE239	7.74	0.25

**Table 7:** Primers designed for site-directed mutagenesis of PS2Aa1.

Primer Id.	Sequence
-Pet100Ps2a_994_FW -Pet100Ps2a_994_RV	5′-TTC CCA AAA GAG TAG NGC CAG GTG GGC ATT A-3′ 5′-TAA TGC CCA CCT GGC NCT ACT CTT TTG GGA A-3′
-Pet100Ps2a_997_FW -Pet100Ps2a_997_RV	5′-CCA AAA GAG TAG GGC NAG GTG GGC ATT ATT T-3′ 5′-AAA TAA TGC CCA CCT NGC CCT ACT CTT TTG G-3′
-Pet100Ps2a_1000_FW -Pet100Ps2a_1000_RV	5′-AAA GAG TAG GGC CAG NTG GGC ATT ATT TTT G-3′ 5′-CAA AAA TAA TGC CCA NCT GGC CCT ACT CTT T-3′
-Pet100Ps2a_1003_FW -Pet100Ps2a_1003_RV	5′-GAG TAG GGC CAG GTG NGC ATT ATT GGT T-3′ 5′-AAC CAA AAA TAA TGC NCA CCT GGC CCT ACT C-3′

## Data Availability

Not applicable.

## Supplementary Materials

The following supporting information can be downloaded at https://www.mdpi.com/article/10.3390/molecules27217262/s1. Figure S1: Cytocidal activities of variants of Parasporin 2 (PS2Aa1) obtained with site-directed mutagenesis to CaCo2 cell lines; Figure S2: Cytocidal activities of variants of Parasporin 2 (PS2Aa1) obtained with site-directed mutagenesis to SW620 cell lines; Figure S3: Cytocidal activities of variants of Parasporin 2 (PS2Aa1) obtained with site-directed mutagenesis to SW480; Figure S4: Cytocidal activities of variants of Parasporin 2 (PS2Aa1) obtained with site-directed mutagenesis to NCM460 cell lines.

## Sample Availability

Not available.
