# Supplementary material for: Site-Directed Mutants of Parasporin PS2Aa1 with Enhanced Cytotoxic Activity in Colorectal Cancer Cell Lines
Source: Molecules. 2022 Oct 26;27(21):7262. doi: 10.3390/molecules27217262 (PMC9658827; doi:10.3390/molecules27217262)
Supplement: Supplementary file 1 [file molecules-27-07262-s001.zip › molecules-1967345-supplementary.pdf]

Site-directed mutants of parasporin PS2Aa1 with enhanced cytotoxic activity in colorectal cancer cell lines

Miguel Orlando Suárez-Barrera[1-3], Lydia Visser[1], Efraín Hernando Pinzón-Reyes[2], Paola Rondón-Villarreal[2], Juan Sebastián Alarcon-Aldana[2], Nohora Juliana Rueda-Forero[2*].

1. Department of Pathology and Medical Biology, University of Groningen, University Medical Center Groningen, University of Groningen, The Netherlands.
2. Facultad de Ciencias Médicas y de la Salud, Instituto de Investigación Masira, Universidad de Santander.
3. Max Planck Tandem Group in Nanobioengineering, institute of Chemistry, Faculty of Natural and Exacts sciences, University of Antioquia.

*Juliana.forero@udes.edu.co

Supplementary information

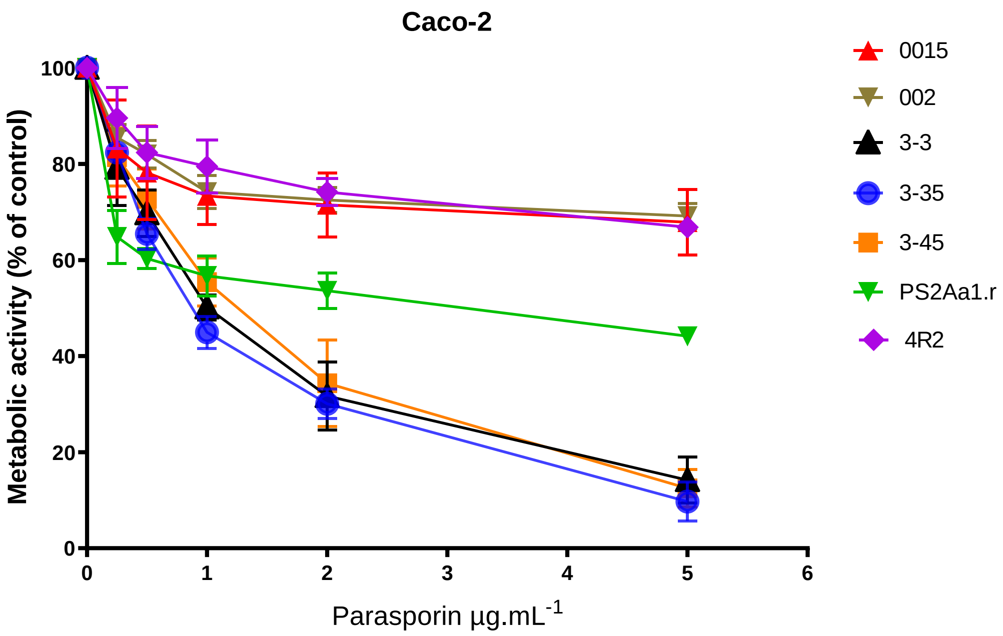

Figure S1: Cytocidal activities of variants of Parasporin 2 (PS2Aa1) obtained with site directed mutagenesis to CaCo2 cell lines. the toxin activated with proteinase K was added (final concentrations, 0.2µg/mL to 5µg/mL).

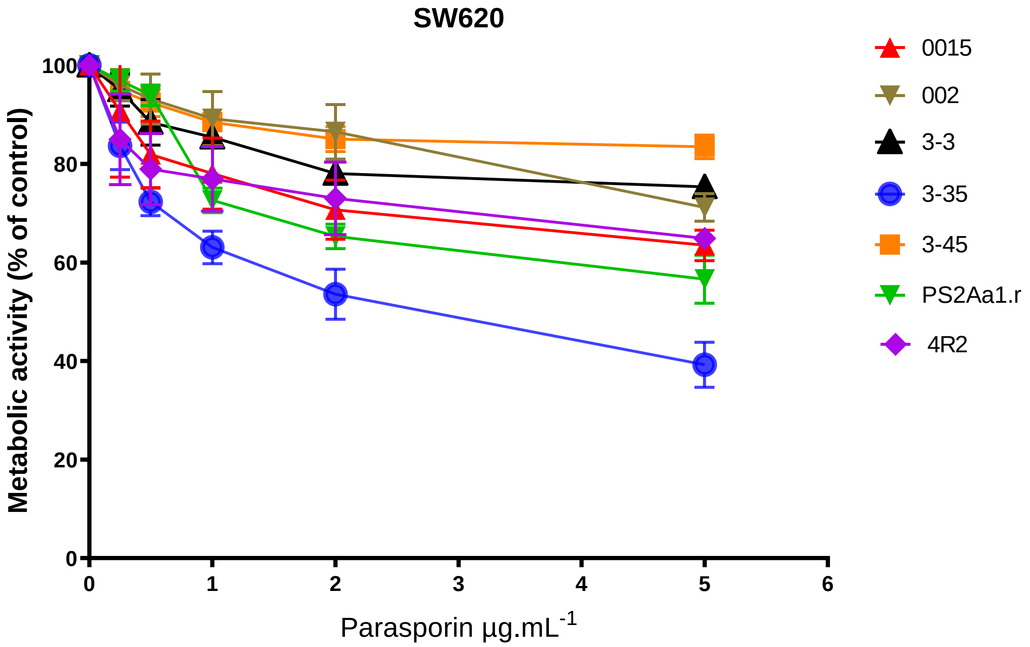

Figure S2: Cytocidal activities of variants of Parasporin 2 (PS2Aa1) obtained with site directed mutagenesis to SW620 cell lines. the toxin activated with proteinase K was added (final concentrations, 0.2µg/mL to 5µg/mL).

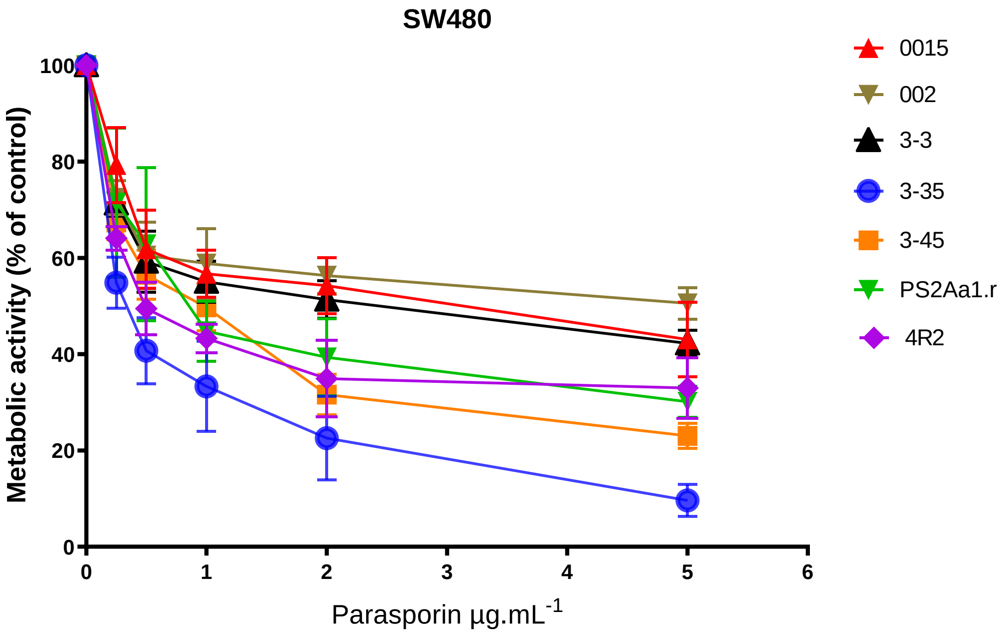

Figure S3: Cytocidal activities of variants of Parasporin 2 (PS2Aa1) obtained with site directed mutagenesis to SW480 cell lines. the toxin activated with proteinase K was added (final concentrations, 0.2µg/mL to 5µg/mL).

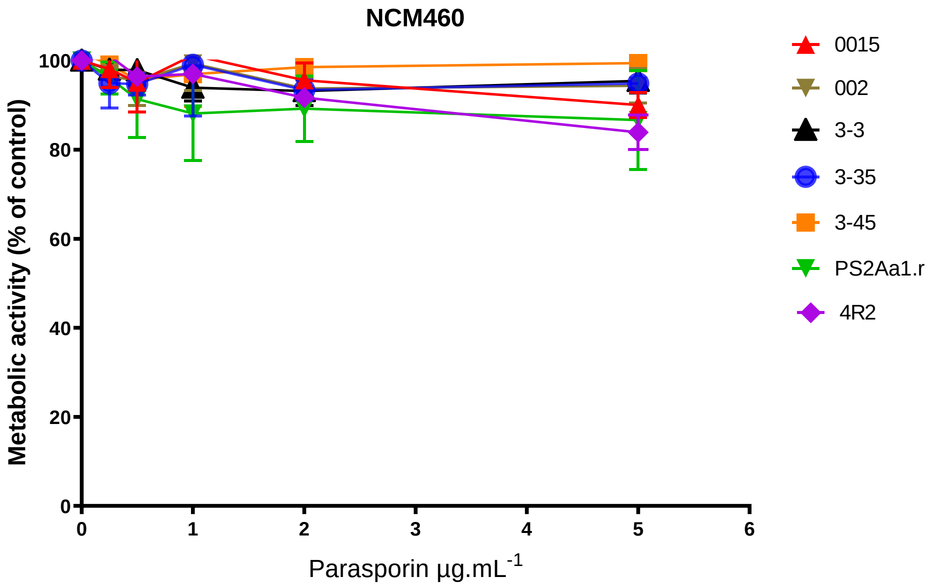

Figure S4: Cytocidal activities of variants of Parasporin 2 (PS2Aa1) obtained with site directed mutagenesis to NCM460 cell lines. the toxin activated with proteinase K was added (final concentrations, 0.2µg/mL to 5µg/mL).
